# Supplementary material for: Impact of COVID-19 on New – onset Type 1 diabetes mellitus: A six-year retrospective review from two Paediatric clinics, Kampala, Uganda
Source: PLoS One. 2026 Feb 5;21(2):e0341145. doi: 10.1371/journal.pone.0341145 (PMC12875443; doi:10.1371/journal.pone.0341145)
Supplement: Attachment — Submitted filename: Response to Reviewers 12.12.2025.doc [file pone.0341145.s002.doc]

**12/December/2025**

**Editorial team**

**PLOS ONE JOURNAL**

**Re: Responses to comments on manuscript entitled** “**Impact of COVID-19 on New – onset Type 1 diabetes mellitus: A six-year retrospective review from two Paediatric clinics, Kampala, Uganda”, submitted to PLOS ONE.**

Dear Editor,

I am pleased to notify you of the responses to the comments recently raised by the reviewer on the above referenced manuscript as shown below:

Reviewers' comments:

Reviewer's Responses to Questions

**Comments to the Author**

1. If the authors have adequately addressed your comments raised in a previous round of review and you feel that this manuscript is now acceptable for publication, you may indicate that here to bypass the “Comments to the Author” section, enter your conflict of interest statement in the “Confidential to Editor” section, and submit your "Accept" recommendation.

Reviewer #1: (No Response)

2. Is the manuscript technically sound, and do the data support the conclusions?

The manuscript must describe a technically sound piece of scientific research with data that supports the conclusions. Experiments must have been conducted rigorously, with appropriate controls, replication, and sample sizes. The conclusions must be drawn appropriately based on the data presented.

Reviewer #1: No

3. Has the statistical analysis been performed appropriately and rigorously?

Reviewer #1: Yes

4. Have the authors made all data underlying the findings in their manuscript fully available?

The [PLOS Data policy](http://track.editorialmanager.com/CL0/http:%2F%2Fwww.plosone.org%2Fstatic%2Fpolicies.action%23sharing/1/010f019a2e6fd7bd-6360ba3d-fa91-46c7-bc19-272eefb9537c-000000/_rxVoMASgv1z78uGwVwTy88sIkGIj0aZpc1DxRsT3B8=235) requires authors to make all data underlying the findings described in their manuscript fully available without restriction, with rare exception (please refer to the Data Availability Statement in the manuscript PDF file). The data should be provided as part of the manuscript or its supporting information, or deposited to a public repository. For example, in addition to summary statistics, the data points behind means, medians and variance measures should be available. If there are restrictions on publicly sharing data—e.g. participant privacy or use of data from a third party—those must be specified.

Reviewer #1: Yes

5. Is the manuscript presented in an intelligible fashion and written in standard English?

PLOS ONE does not copyedit accepted manuscripts, so the language in submitted articles must be clear, correct, and unambiguous. Any typographical or grammatical errors should be corrected at revision, so please note any specific errors here.

Reviewer #1: Yes

6. Review Comments to the Author

Please use the space provided to explain your answers to the questions above. You may also include additional comments for the author, including concerns about dual publication, research ethics, or publication ethics. (Please upload your review as an attachment if it exceeds 20,000 characters)

Reviewer #1: Response to response:
Introduction:
1. there are many more papers that need to be cited ( for example <https://doi.org/10.1002/jmv.27996>, <https://doi.org/10.2337/dc21-0969>, <https://doi.org/10.1155/2023/4580809>, <https://doi.org/10.1016/j.dsx.2021.02.009>)
Response: These have been cited in the last sentence of the first paragraph of the discussion section. References 12-15.

New Response: great


2. an explanation is needed for how these two clinics are meant to be stand ins for the entire country.
Response: These two pediatric diabetic clinics at Mulago and Nsambya hospitals are the largest main treatment and referral centres for childhood diabetes in Uganda. They treat the majority of Ugandan pediatric T1DM and are referral centres for T1DM for the whole country. The statement on findings and conclusions has been clarified that the findings of this study were at the two clinics in Kampala, Uganda.


New Response: you do not address my main commentary. Until this is addressed, I cannot allow this paper to proceed. How can these very few patients be a stand in for the whole country? There should be thousands of kids of DM and you access a few hundred. If my math in the previous review was incorrect, please explain to me why. You don't have to do it in the paper itself if my argument makes no sense, but you have to address it.

**New author’s Response**: The two paediatric diabetic clinics at Mulago and Nsambya Hospitals are the largest main tertiary treatment and referral centres for childhood diabetes in Uganda, located in the capital city, Kampala. They are the hubs where other Type 1 Diabetes clinics refer their patients for care. Overall, approximately 5000 children and adolescents are registered with T1DM in Uganda, and ~ 800 (16%) children and adolescents are registered between these two clinics. In Uganda, during the COVID 19 era, the COVID 19 screening was mostly concentrated in urban settings mostly in Kampala and the surrounding areas as compared to rural areas. These two clinics are located in the urban setting at the heart of Kampala, and these are the areas where critical data regarding COVID 19 was registered. These clinics were also accessible to the researchers and have quality data that could be used for this research thus the choice of the two clinics. Due to limited funding and resources for this study, we couldn’t assess the incidence of T1DM in Ugandan children and adolescents in the whole country, but a National-scale study was recommended to address this gap as stated in the second last sentence under discussion section of the manuscript.

Methods:
1 until what age are these patients seen/included in the study?
Response: From ages 6months to 19years. This was already mentioned in the abstract in the methods section, and has been re emphasised in the first paragraph of the study design under methods section.


New Response: great

2. there's lots of unnecessary details about how the clinics are run but no explanation for where else patients might be going. Is there selection bias in who comes to the clinics?
Response: There is no difference in the sociodemographic characteristics of children with T1DM who are seen at either Mulago or Nsambya diabetic clinics. Services provided are similar at both clinics and free of charge under the support of the Government of Uganda and World Diabetes Foundation’s Changing diabetes in children (CDiC) project. A few children with T1DM seen at Lubaga Hospital, and others diagnosed from other hospitals in the country are linked to these two clinics for treatment support and follow up.


New Response: My question was not about the difference between the two clinics but between patients who are seen in these clinics and those who are not. Harking back to my main point, thousands of Ugandan kids are unaccounted for here. Who sees them? Are they different? How?

**New author’s response**: Mulago and Nsambya Diabetic clinics are the largest tertiary treatment centres for children and adolescents with diabetes in Uganda. They are also referral centres for other clinics in the country however, the patients seen at these clinics are similar in characteristics to those seen elsewhere. These two clinics are run by paediatrician/ endocrinologist, medical residents, interns and/ medical officers. Mulago hospital is public hospital that provides free health care services to all the patients they see while Nsambya is a private not for profit hospital that provides free insulin and diabetes supplies however other services at the hospital like admissions and blood tests are for pay. Other children elsewhere in the country with diabetes are mainly seen at public regional hospitals in 44 facilities by clinicians including medical officers, clinical officers and some trained nurses. The average number of children and adolescents registered with diabetes at the different health facilities is ~ 100 as compared to about ~400 at each clinic at Mulago and Nsambya.

An explanation on where the other children with diabetes are seen in Uganda has been added as the last paragraph of the study setting section under materials and methods section of the manuscript

Results:
1. main finding of the paper is not statistically significant, ie is not really a finding
Response: This has been clarified that it was not statistically significant


New Response: Great


Discussion:
Paragraph one is not entirely true in view of above
Response: This has been clarified that much as there was an observed increase in the trend of T1DM, it was not statistically significant.


New Response: Great

7. PLOS authors have the option to publish the peer review history of their article ([what does this mean?](https://track.editorialmanager.com/CL0/https:%2F%2Fjournals.plos.org%2Fplosone%2Fs%2Feditorial-and-peer-review-process%23loc-peer-review-history/1/010f019a2e6fd7bd-6360ba3d-fa91-46c7-bc19-272eefb9537c-000000/6qUX5XyEDK46_vkiV_z6AG6gkHboLFh0hjnsKO48zjI=235)). If published, this will include your full peer review and any attached files.


If you choose “no”, your identity will remain anonymous but your review may still be made public.


**Do you want your identity to be public for this peer review?** For information about this choice, including consent withdrawal, please see our [Privacy Policy](https://track.editorialmanager.com/CL0/https:%2F%2Fwww.plos.org%2Fprivacy-policy/1/010f019a2e6fd7bd-6360ba3d-fa91-46c7-bc19-272eefb9537c-000000/cAwKuD3PBQPIgEUEV2DT0M6LyvhOK3SYA3Hq4KEvLVQ=235).

Reviewer #1: **Yes:** Renee Bargman

Yours Thankfully

…………………………….

ROSEMARY AHABWENKI

MBChB, MMed

Department of peadtrics and child health

Makerere university

Email: [rosieahabs@gmail.com](mailto:rosieahabs@gmail.com)
